# Characterization of New PEEK/HA Composites with 3D HA Network Fabricated by Extrusion Freeforming

**DOI:** 10.3390/molecules21060687

**Published:** 2016-05-26

**Authors:** Mohammad Vaezi, Cameron Black, David M. R. Gibbs, Richard O. C. Oreffo, Mark Brady, Mohamed Moshrefi-Torbati, Shoufeng Yang

**Affiliations:** 1 Engineering Materials Group, Faculty of Engineering and the Environment, University of Southampton, Southampton SO17 1BJ, UK; mv1y11@soton.ac.uk (M.V.); m.m.torbati@soton.ac.uk (M.M.-T.); 2 Bone and Joint Research Group, Centre for Human Development, Stem Cells and Regeneration, Faculty of Medicine, University of Southampton, Southampton SO16 6YD, UK; crmb1e12@soton.ac.uk (C.B.); dmg1e12@soton.ac.uk (D.M.R.G.); richard.oreffo@soton.ac.uk (R.O.C.O.); 3 Invibio Ltd., Thornton-Cleveleys, Lancashire FY5 4QD, UK; mbrady@invibio.com

**Keywords:** polyetheretherketone (PEEK), additive manufacturing (AM), extrusion freeforming, compression molding, bioactive PEEK/HA composite, porous PEEK

## Abstract

Addition of bioactive materials such as calcium phosphates or Bioglass, and incorporation of porosity into polyetheretherketone (PEEK) has been identified as an effective approach to improve bone-implant interfaces and osseointegration of PEEK-based devices. In this paper, a novel production technique based on the extrusion freeforming method is proposed that yields a bioactive PEEK/hydroxyapatite (PEEK/HA) composite with a unique configuration in which the bioactive phase (*i.e.*, HA) distribution is computer-controlled within a PEEK matrix. The 100% interconnectivity of the HA network in the biocomposite confers an advantage over alternative forms of other microstructural configurations. Moreover, the technique can be employed to produce porous PEEK structures with controlled pore size and distribution, facilitating greater cellular infiltration and biological integration of PEEK composites within patient tissue. The results of unconfined, uniaxial compressive tests on these new PEEK/HA biocomposites with 40% HA under both static and cyclic mode were promising, showing the composites possess yield and compressive strength within the range of human cortical bone suitable for load bearing applications. In addition, preliminary evidence supporting initial biological safety of the new technique developed is demonstrated in this paper. Sufficient cell attachment, sustained viability in contact with the sample over a seven-day period, evidence of cell bridging and matrix deposition all confirmed excellent biocompatibility.

## 1. Introduction

There has been a trend in recent years to develop polyetheretherketone (PEEK)-based medical devices due to PEEK’s excellent cell biocompatibility and desirable mechanical properties such as strength, and elastic modulus; comparable to cortical bone [1,2]. Medical grade PEEK-OPTIMA has been developed to meet the US Food and Drug Administration’s (FDA) requirements and has been used in multiple clinical applications including spinal cage fusion and crani-omaxillofacial reconstruction [3,4]. Calcium phosphates including hydroxyapatite (HA) and β-tricalcium phosphate (β-TCP), or Bioglass, are utilized as a composite filler to produce PEEK compounds with potential for osseointegration [5,6]. Recent study on nano-TiO_2_/PEEK biocomposites [7] revealed that pseudopods of osteoblasts preferred to anchor at areas where nano-TiO_2_ was present on the surface. In addition, the addition of nanocalcium silicate into PEEK can significantly promote cell attachment, proliferation, and spreading compared with PEEK [8]. In addition, incorporation of porosity into PEEK has been realized as an effective method to improve bone apposition [9,10]. Alternatively, surface modification has been used to enhance the mechanical and biological properties of PEEK [11,12,13,14]. Titanium and HA coated PEEK has also shown much higher bone-to-implant contact ratio than the pure PEEK implants [15,16]. A review of strategies to improve the bioactivity of PEEK has been reported recently [5].

Different processing methods such as compounding and injection molding [17,18,19,20,21], compression molding [22,23,24,25], cold press sintering [26,27,28] and selective laser sintering (SLS) [29,30,31,32] have been used to produce bioactive PEEK/HA and β-TCP composites. Functionally graded PEEK/HA biocomposites can also be produced by layer-by-layer casting method [33].

Compression molding is low-cost, suitable for high-volume production of very dense PEEK compounds and, more critically, tailored porosity can be achieved by the addition of a fugitive particle (e.g., sodium chloride) into the compound that is subsequently leached out by soaking into a solvent, post molding [34]. SLS, a type of powder-based additive manufacturing (AM) technology, is capable of fabricating bioactive PEEK/HA structures with very complex architecture, permitting greater design freedom. The SLS technique has been hampered by difficulty in loading the quantity of bioactive filler beyond 22% by volume, and exceeding porosity beyond 70 vol. %–74 vol. % [28].

The methods of bioactive PEEK processing discussed earlier, do not permit control of distribution of the bioactive phase within the PEEK matrix. This limitation results from reliance of these techniques on mixing PEEK with bioactive material in powder or granular forms. In addition, the wide range of physical properties of these particles (*i.e.*, size, shape, and density) typically hinders efficient and consistent mixing. A new technique is proposed in this study that provides greater control on incorporation of bioactive materials into PEEK. The proposed technique integrates extrusion-based AM and compression molding processes and provides new possibilities to produce both bioactive PEEK compounds and porous PEEK structures with enhanced biological performance. Furthermore, this approach enables designers to control precisely the distribution of bioactive phase within the PEEK matrix and hence tailor biological and mechanical properties of the final composite. Moreover, both bioactive phase and PEEK matrix are fully interconnected, which is superior to existing microstructural designs. The technique is versatile, such that a range of bioactive materials such as Bioglass, β-TCP, *etc.* with different rates of biodegradation can be used and the interconnected bioactive network can be fully absorbed *in vivo*, leaving 3D interconnected channels for further in-growth and proliferation. Thus, a 3D locked bone/PEEK structure could be achieved *in vivo*, which can dramatically improve implant fixation compared with existing techniques.

Techniques such as particulate leaching, heat and compression sintering, micromachining, sulfonation treatment, and SLS have been used to make porous PEEK for medical applications [9,35]. The authors reported successful low-cost 3D printing of porous PEEK structures with controlled pore size using filament-based extrusion AM process [36]. Formation of pores within PEEK results in a physical form that more closely replicates natural tissue, permitting cellular infiltration and integration with surrounding tissue (preventing device migration). Particulate leaching has been shown to be a flexible and economic platform among currently available porous PEEK manufacturing techniques but has less control on micro/macrostructure (e.g., porosity and architecture), lacks versatility and still suffers from limitations such as manual intervention, and inconsistency. In addition to bioactive PEEK compounds, the proposed technique can be utilized to produce porous PEEK with fully interconnected pores and controlled porosity which will be discussed further. To the best knowledge of the authors, this is the first report of PEEK/HA composite displaying 100% interconnectivity of the bioactive HA network phase. Further, this is also the first report on fully interconnected porous PEEK structure with controlled pore size, porosity, and channel direction and distribution.

In this paper, the results are presented first in Section 2 and discussed in Section 3, while the experimental methods are introduced in Section 4.

## 2. Results

### 2.1. 3D Printed HA Scaffolds

**Figure 1:**
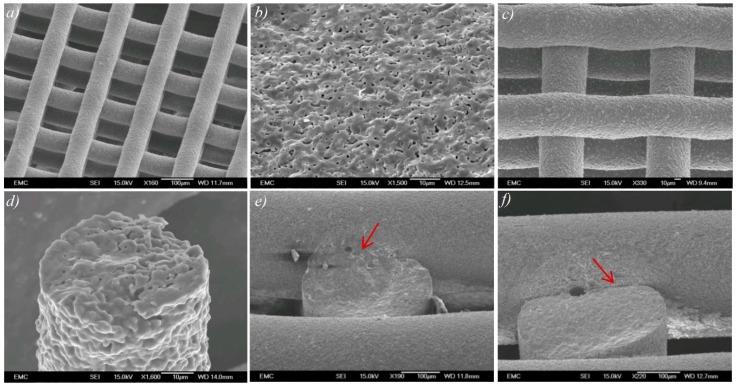
(**a**) A typical sintered hydroxyapatite (HA) scaffold with uniform microstructure and macroporosity; (**b**) magnified image of fractured surface of HA filament with micropores; (**c**) 3D printed HA scaffold with filament deformation upon deposition due to high solvent content in paste; (**d**) magnified view of a 50 μm HA filament; (**e**) strong inter layer bonding due to sufficient solvent content in HA paste; and (**f**) weak layer bonding due to insufficient solvent content. The red arrow in the images (**e**,**f**) shows bonding formed between layers in the slurry state.

HA scaffolds with a range of filament and pore sizes were printed using the bespoke developed 3D printer. Through control of the printing parameters such as solvent content in the paste, nozzle size, paste deposition speed, and build layer thickness it was possible to determine the microstructure of the scaffolds. The printed scaffolds were highly uniform (Figure 1a), and the production process was consistent and repeatable. Figure 1b shows a magnified view of a filament fracture surface in which sintering-induced pores within the range of 1–5 μm are evident. Both macroporosity of scaffold (spacing in Figure 1a) and microporosity of filaments (pores in Figure 1b) are controllable in solvent-based extrusion freeforming: macroporosity of scaffold by computer design and microporosity of filaments via alternation in sintering temperature and dwell time [37].

Macroporosity and microporosity of HA scaffolds of various pore and filament sizes were measured using Archimedes’ method, as shown in Table 1. The macroporosity could be controlled in a wide range, from 33% to nearly 70% in the four samples, with the macroporosity of sample 400.250 being significantly smaller than the others due to its larger filament size. Actually, the macroporosity range could be further increased to 20%–80% by changing the filament diameter and/or the filament distance. However, too low or too high macroporosity might not be ideal for this application. If the porosity is too low (more HA phase), it will be difficult to infiltrate enough PEEK to provide sufficient mechanical strength to the composite. If the porosity is too high (too little HA) there could be insufficient bioactive phase after infiltration. The microporosity calculated across all samples lies within the range of 7%–10% with little variability, showing consistency in the properties of the material used to produce each sample and minimal presence of pores within the filaments.

**Figure 2:**
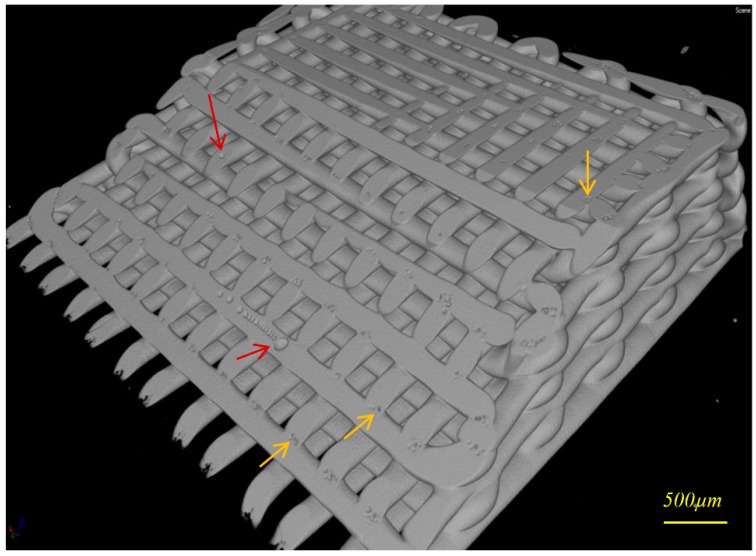
Computed tomography (CT) image of a typical 3D printed HA scaffold sectioned by horizontal and oblique plans, micro-air bubbles inside filaments (red arrows), and in filaments’ welding areas (yellow arrows).

Figure 2 shows a CT image taken of an oblique cross section of a typical 3D printed HA scaffold in which air bubbles/voids are seen. Air bubbles inside extruded filaments (red arrows) are produced during paste preparation (e.g., paste stirring, solvent evaporation, and loading the paste into the syringe).

### 2.2. Bioactive PEEK/HA Composite

CT analysis of PEEK/HA composites with the proportion of HA ranging from 41 vol. % to 78 vol. % resulted in an average of 1.5 vol. % air within the PEEK matrix (Table 2). A small variation (up to approximately 5%) in HA percentage volume calculated by the image processing application (VG Studio Max 2.1, Volume Graphics GmbH, Heidelberg, Germany) for the samples with the same HA filament/pore size in Table 2 proves repeatability and accuracy of the 3D printing process. No specific correlation between pore size and air bubbles formation was found, with 400 μm pore size resulting in the greatest volume of air (3.2%). However, using HA scaffolds with larger pore size at size 700 μm resulted in formation of more air bubbles (up to approximately 5 vol. %) within PEEK matrix.

**Figure 3:**
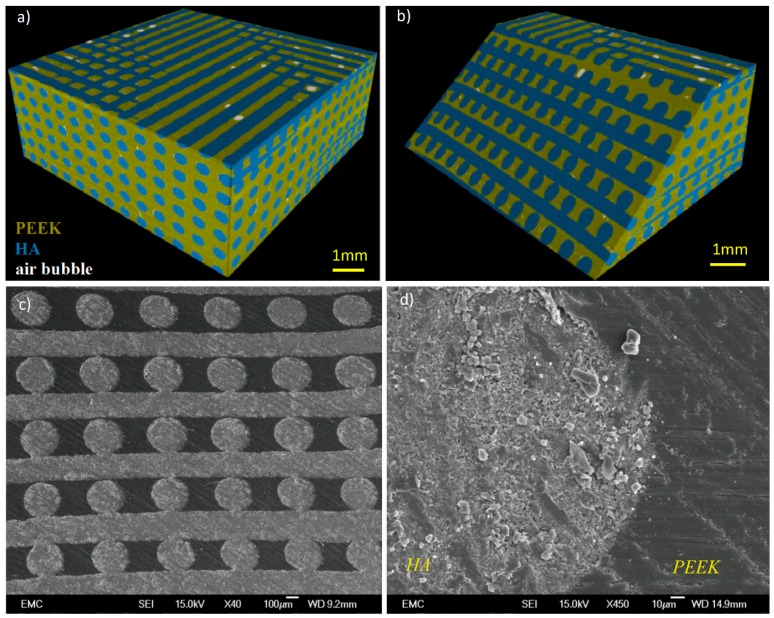
(**a**) 3D image constructed from CT scan of a PEEK/HA composite with: HA filament size 400 μm/pore size 400 μm, HA 58%, air bubble 2.4%, PEEK 39% (total volume: 221 mm^3^); (**b**) oblique section view; (**c**) scanning electron microscope (SEM) image of a typical PEEK/HA composite, scaffold size 10 mm × 10 mm × 3 mm, HA scaffold filament size: 250 μm, pore size 200 μm, molding temperature: 400 °C, dwelling time: 20 min, heating rate: 20 °C/min, static pressure: 0.39 MPa; and (**d**) close view of HA/PEEK interface.

Figure 3 illustrates representative 3D CT and SEM images of typical PEEK/HA composites produced successfully through compression molding using static loading [38]. As seen in the CT images of various cross sections, the majority of air bubbles are located in the upper region in the images, which corresponds to the bottom surface of the mold, where air release would be most likely to be impeded. HA scaffold with a pore size of 200 μm is fully infiltrated by PEEK in both vertical (infiltration depth was 3 mm) and lateral directions, while maintaining the HA network structure and uniformity (Figure 3c). Both bioactive phase and PEEK matrix are fully interconnected, which is superior to existing techniques [20,21,22,23,24,25,26,27,28].

### 2.3. Porous PEEK Structure

**Figure 4:**
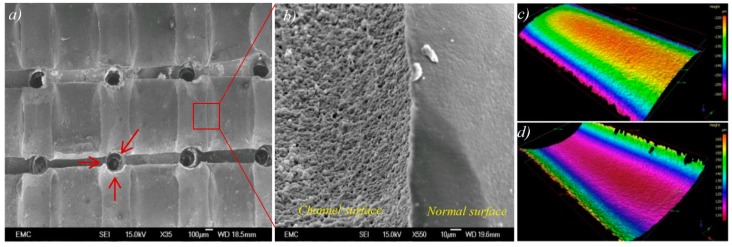
(**a**) Top view of the porous PEEK sample showing interconnectivity of the channels, red arrows indicate crossed channels; (**b**) magnified view from surface of a channel within a typical porous PEEK; and (**c**,**d**) representative 3D surface height maps are shown for 400 μm HA filament and the channel produced with the use of a same size HA filament: (**c**) profile of HA filament surface and (**d**) profile of PEEK channel surface.

Porous PEEK samples were produced by soaking the PEEK/HA composite into hydrochloric acid (HCl) solution for 72 h. HA filaments were dissolved in HCl, resulting in hollow channels suitable for cell attachment, infiltration and proliferation. Critical material properties for orthopedic tissue engineering include: (i) sufficient pore interconnectivity for cell infiltration and perfusion of nutrients; and, secondly, (ii) adequate pore size to facilitate vascularization, while still affording sufficient mechanical strength [35]. The channels could help the alignment and differentiation of cells [39]. The use of extrusion freeforming in this research work permitted excellent control on pore size and interconnectivity (Figure 4a), which is necessary for bone-ingrowth.

Further analysis of the SEM images revealed that the replica of the surface of HA filaments is produced onto the surface of channels in PEEK following HA removal (Figure 4b). Optical profilometry was used to measure surface roughness of a typical 400 μm HA filament and the replica in a channel in the PEEK. Figure 4c,d shows the 3D surface height maps captured from the surface of an HA filament, and its replica in PEEK, respectively. As it was very difficult to prepare samples with exactly the same filaments and channels, which requires very precise sample marking before and after infiltration, a direct comparison was not conducted. However, it is reasonable to assume that the filaments produced in the same batch have very similar surface morphology. Both the surface of HA filament and the surface of the channel in PEEK (produced from the same batch, and the same size of HA filaments) had the same average surface roughness (Ra) of 0.4 μm, with standard deviations of 37 nm and 54 nm, respectively. This demonstrates that there is excellent contact between PEEK and HA surfaces within the PEEK/HA composite.

### 2.4. Mechanical Properties of the PEEK/HA Biocomposites

These composites can be used for either load bearing or non-load bearing applications such as cranial maxillofacial (CMF) plate, *etc.* The initial intention of this research work was to highlight extrusion freeforming technology as having potential application in production of these new PEEK/HA composites. Whilst the addition of bioactive materials to PEEK offers an efficient system to engineer implants with tailored biomechanical properties, it may result in reduced strength of the implant, decrease in fracture energy and the brittleness of the composite materials [34]. Mechanical behavior of PEEK/HA composites has also been studied extensively [17,18,19,40,41]. Elastic modulus, compressive strength and micro indentation hardness is enhanced by increasing HA loading, while tensile strength, toughness and strain to failure is decreased. Loading PEEK with 40 vol. % HA has been shown to decrease the ultimate tensile strength (UTS) of the PEEK by 45%, to 44 MPa [2]. Assessment of mechanical properties would be an important metric of performance of these new biocomposites for potential compressive load bearing applications such as spinal cage fusion, *etc.*

**Figure 5:**
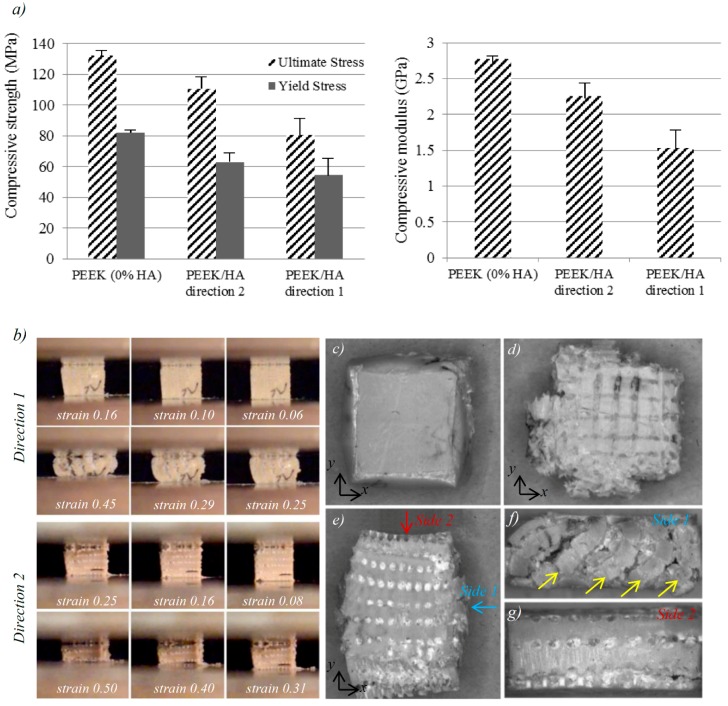
(**a**) Compressive elastic modulus, ultimate and yield strength determined for PEEK and PEEK/HA specimens compressed in different directions; (**b**) sequential images of PEEK/HA biocomposites at size 6 × 6 × 6 mm^3^ with 40 Vol% HA during compression in different directions; and (**c**–**g**) optical images of the samples after compression test: (**c**) top view of unfilled PEEK sample; (**d**) top view of PEEK/HA compressed in direction 1; (**e**) top view of PEEK/HA compressed in direction 2; and (**f**,**g**) front and side view of PEEK/HA compressed in direction 2. The yellow arrows in the image (**f**) show the plastic flow under pressure.

Figure 5a shows the results of compression tests on the biocomposites with average HA content of 40 vol. % tested in two different directions (The directions 1 and 2 are defined in experimental section) and compared with unfilled PEEK samples (*i.e.*, 0% HA). Sequential images of the PEEK/HA biocomposites during compression in different directions can be seen in Figure 5b. Table 3 shows a comparison between compressive properties of the unfilled PEEK, the PEEK/HA composites with 40% HA, and human cortical bone (data for human cortical bone taken from [42]).

**Figure 6:**
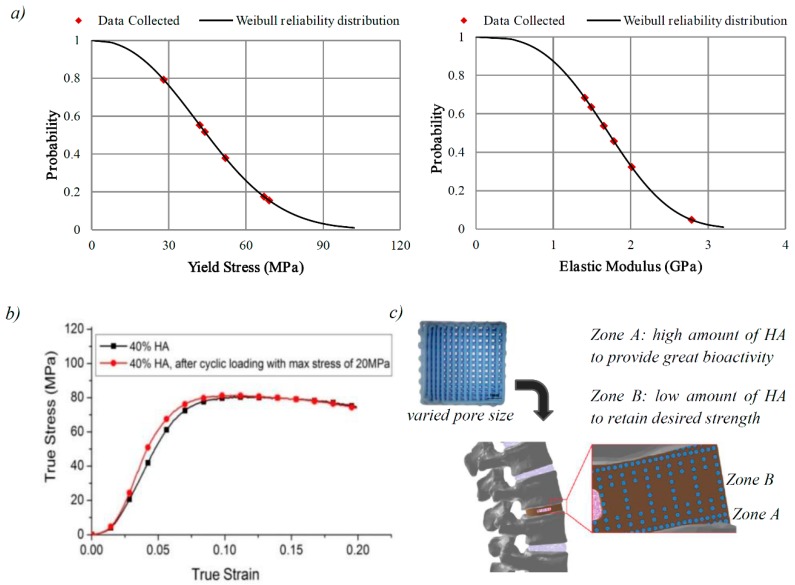
(**a**) Weibull reliability distribution for yield stress and elastic modulus; (**b**) compressive stress–strain plot for PEEK/HA composites with 40 vol. % HA before and after one million cyclic loading in direction 1; and (**c**) schematic of the use of 3D printed hierarchical HA scaffold with computer-controlled varied spacing suitable to make functionally graded PEEK/HA composites for spinal cage fusion.

Weibull reliability distribution curves for yield stress and elastic modulus were plotted for samples tested in direction 1 (Figure 6a). The calculation method is presented in the Suppementary Materials. According to Weibull reliability curves, about 93% of the samples had compressive yield strength of above 20 MPa, and elastic modulus above 0.8 GPa. Taking this into consideration, another set of samples with the same HA content was prepared and subjected to compressive-compressive cyclic loading with maximum and minimum stress of 20 MPa and 2 MPa for 1 million cycles. Afterward, the samples were subjected to compression to failure test in order to evaluate cyclic loading endurance of the biocomposites. No significant difference in compressive strength was observed between the cyclic loaded samples and the normal samples (Figure 6b). The cyclic loaded samples had relatively similar yield and ultimate strength, but smaller yield strain, and higher elastic modulus of 1.93 GPa. Previous studies on tension–tension fatigue behavior of the PEEK/HA composites prepared using compression molding showed that spherical HA particles in PEEK/HA composites could debond from the PEEK matrix during long-term cyclic loading due to the poor interfacial adhesion [18]. To improve PEEK-HA interface, well-dispersed HA nanoparticles [20,21,43,44] can be used although agglomeration of HA nanoparticles becomes an issue when the loading is over 10 vol. % [43].

### 2.5. Biological Performance of the PEEK/HA Biocomposites

**Figure 7:**
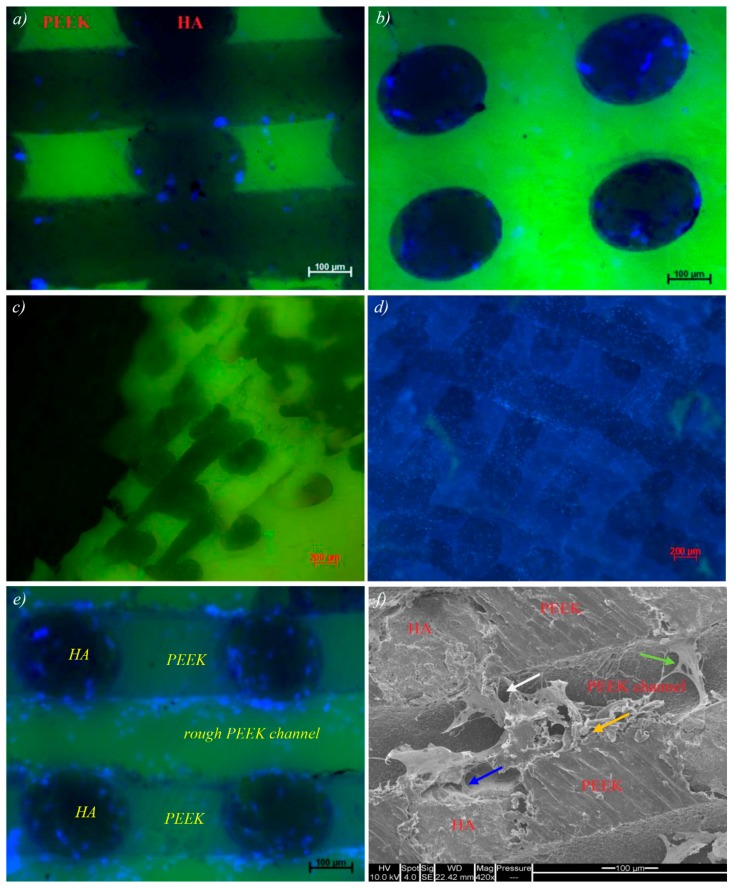
(**a**–**d**) Cell attachment on PEEK/HA composite, DAPI nuclear staining (4′,6-diamidino-2-phenylindole, blue) indicates the wide scale presence of adherent cells throughout the composite substance. Strong scaffold green-channel autofluorescence competes with Cell tracker Green signal reducing the effectiveness of this assay; (**e**) cell attachment on HA and rough PEEK channels within the composite; and (**f**) SEM imaging reveals the cell–surface interaction and adhesion at HA surface (blue arrow), PEEK/HA boundary (white arrow) and the interface of PEEK surface roughness variations (orange arrow).

*In vitro* biocompatibility of representative extrusion free-formed PEEK/HA samples was assessed using standard cell viability protocols based on guidelines set out in ISO 10993. Viable, adherent cells were visible as assessed under fluorescent microscopy after seven days in culture. Cell viability at this time point was confirmed by incubation with Cell Tracker Green, the presence of non-viable cells visualized by Ethidium Homodimer incubation and uptake. In Figure 7a–d, adherent Human bone marrow stromal cells (hBMSC) are seen extensively over the surface of the PEEK/HA samples confirming a broad degree of cytocompatibilty across multiple samples, cultured over a biologically relevant time period of seven days. DAPI stained cell nuclei, shown in blue, confirmed cell locality to regions of PEEK/HA interface. Non-quantitative observation suggested a preference in cell adhesion to HA surface areas over PEEK regions within individual samples. The majority of non-viable, non-adherent cells were removed during the PBS rising, which preceded media changes, and incubation with Live/Dead indicators. All remaining, adherent cells were confirmed viable by the absence of observed 617 nm wavelength emission after incubation with Ethidium Homodimer.

Figure 7e shows the result of *in vitro* test on a sample with HA and rough PEEK channels (following removal of the HA phase). The PEEK channels were simply obtained on the surface after cutting the samples using diamond cutter as HA-PEEK bonding could not bear the high shear stress induced by high speed cutting process. SEM imaging helps reveal the cell–surface interaction and adhesion at: (a) HA surface; (b) PEEK/HA boundary; and (c) Interface of PEEK surface roughness variations. The green arrow in Figure 7f illustrates a bridging effect whereby proliferating cells appear to anchor and branch from the composite HA network and attach to either the rough PEEK channel surface or HA phase on the other side. 

## 3. Discussion

### 3.1. 3D Printed HA Scaffolds

The key feature of the developed extrusion-based AM method is its unique nozzle design, and paste formulation which allows very uniform pastes to be made quickly with excellent extrudability and less nozzle jamming during extrusion. Lewis and co-workers, leaders in extrusion-based 3D direct writing, reported printing features as fine as 250 nm for sol-gel inks [45], ~10 μm for hydrogel inks [46,47], and ~1 μm for polyelectrolyte inks [48], while they have reported the minimum feature size of ~200 μm for ceramic inks [49,50]. Minimum feature size is limited for ceramic inks as these inks either experience nozzle jamming (or clogging) or need exceedingly high pressure to impel ceramic ink to flow through a fine nozzle [51,52]. HA scaffolds with filaments as fine as 50 μm could be printed in this research work for the first time using the developed extrusion freeforming device by carefully tuning paste formulation for optimal paste consistency and using a unique nozzle design with minimum die land.

The quality of the lattices produced depended largely on paste rheology and the subsequent paste extrusion settings. The viscosity of the paste when loaded into the extrusion syringe affects the formability and more importantly shape sustainability of the filament that is extruded during the print procedure; a paste with too low viscosity (high solvent content) would result in a filament that is less able to retain its shape, and more likely to deform upon settling on the built layer (Figure 1c). On the other hand, solvent content in the prepared HA paste must be sufficient for formation of a strong weld between layers. If solvent content is too low, resulting in faster extrudate drying and insufficient weld formation, it can affect mechanical strength of the 3D printed scaffold, which is critical to sustain subsequent compression molding. Figure 1e demonstrates a sample printed using paste with adequate solvent content in which bonding formed between layers in the slurry state is retained after sintering, and provides a strong bridge between layers. In Figure 1f, the weld formed in the slurry state lacks sufficient mechanical strength for compression molding as the HA paste does not contain sufficient solvent.

CT analysis of 3D printed scaffolds revealed that in addition to sintering-induced micropores there are two kinds of air bubbles/voids produced in the scaffolds: air trapped within filaments, and voids at the point of welding between the extruded filaments. These micro air bubbles/voids can affect the structural integrity of scaffolds, and it is important to minimize them to enable compression molding to be performed without damaging the HA network.

It was found that it is important to initialize the printing by dumping a few centimeters of filament until a stable extrusion started to minimize air bubbles inside extruded filaments. This is because during the paste loading stage, in which the paste is manually pushed into the syringe, the recess of the syringe is not fully loaded and air pockets can be trapped. During the initialization of extrusion, the trapped air is expelled and then extrusion became stable. The finishing of the initialization stage could be determined by reading the extrusion force, which fluctuates during initialization stage but becomes stable later. After paste extrusion reaches a relatively steady state, scaffold printing may be commenced with a reduction of air bubbles within the filaments. In addition, selection of a finer nozzle in the initial stage necessitates greater extrusion pressure, and thus more chance for air in the HA paste to be expelled through the outlet in this stage. The mechanism of micro-bubble formation between filaments in the welding zone (yellow arrow in Figure 2) is yet to be understood.

### 3.2. Bioactive PEEK/HA Composite

Control factors such as mold temperature, pressure, loading method and dwell time need to be optimized for infiltration of PEEK into the fragile HA scaffolds in compression molding. Based on the reported positive effects of using high temperatures on both shear viscosity of PEEK and mechanical properties of the resulting compression molded parts [53,54] with no adverse effect on biocompatibility *in vivo* [54], a mold temperature of 400 °C was selected in this study. While mold temperatures of 400 °C guarantee minimum viscosity, caution must be exercised when working at temperature in excess of 380 °C as there is a risk of thermal oxidation [55]. Potential for oxidation can be decreased by a reduction in dwell time spent at the target temperature; such optimization of dwell time is crucial. From experiments, it was determined that 20 min was the optimal dwell time for compression molding of PEEK at 400 °C, permitting adequate melt flow to fill the lattice structures with no apparent polymer degradation [38].

The 3D printed HA scaffolds are relatively fragile and molding pressure must be carefully regulated to permit flow of molten PEEK and perfusion of fine pores without resulting in fracturing of the HA network. A series of molding pressures were tested and CT analysis was used to detect damage on HA scaffolds after the compression molding. Through experimentation, the optimal pressure to ensure full infiltration of a scaffold at size 10 × 10 × 3 mm^3^ with pore size above 200 μm, without resulting in damage, was determined to be in the region of 0.39 MPa [38]. This optimal pressure is similar to that used by other researchers in the compression molding of carbon fiber reinforced-PEEK (CFR-PEEK) composites [56,57,58]. Luo *et al.* [56] tested pressures ranging from 0.5 to 2.0 MPa and found that 0.5 MPa is the most suitable pressure for making 3D CFR-PEEK composites prepared by 3D co-braiding and compression molding techniques. Mrse and Piggott [58] employed 0.4 MPa pressure for the preparation of AS-4 carbon-fiber reinforced PEEK using lay-up followed by compression molding process to avoid fiber damage.

The good interface achieved between HA filaments and PEEK matrix shown in Figure 3d proves that the molding temperature and pressure were selected appropriately. In CFR-PEEK, good bonding between PEEK matrix and carbon fibers is critical and determines the overall strength of the composite as it enables load transfer from PEEK to carbon fibers [59]. Whilst a good interface between HA filaments and PEEK matrix is realized, the interfacial bonding might not be as important as CFR-PEEK since the brittle HA filaments are used for providing bioactivity, rather than load bearing. In contrast, HA scaffold volume fraction, and filament diameter/orientation could be pivotal in determining the mechanical properties of the final composite.

### 3.3. Porous PEEK Structure

As mentioned previously, conventional techniques such as particulate leaching has poor control on porosity, and suffers from limitations such as inconsistency and the requirement for manual intervention. Using the proposed technique, porous PEEK can be easily produced with much higher control level on porosity and enhanced reproducibility, a key requirement in production of medical devices. Furthermore, the technique is a low-cost process in comparison with the SLS process, while affording greater control of pore size (the minimum achievable pore size in SLS is currently ~500 μm due to limitation in de-powdering). Therefore, while SLS as an AM technology has great control on pore size and architecture, its use in clinical applications is currently limited to those in which pore sizes above 500 μm are required. The limitation in minimum pore size makes it difficult to design an implant with desired strength as well. Moreover, the very low recycle rate of PEEK powder makes SLS an extremely expensive and non-eco-friendly process. In contrast, with the use of the proposed technique a wide range of pore sizes (200–1000 μm) is achievable. This makes the technique a versatile approach, enabling formation of porous PEEK which can be tailored to specific biomechanical requirements of a variety of clinical applications.

As seen in Figure 4b, the replica of the surface of HA filaments is produced onto the surface of channels in PEEK following HA removal. This demonstrated that despite the high viscosity of molten PEEK, micropores on the surface of the HA filaments could be filled by PEEK through compression molding. This guarantees a good interface between PEEK and HA filaments in PEEK/HA composite and more critically the rough surface of channels within porous PEEK can enhance bone cell attachment, proliferation, and differentiation [60,61]. Kumar *et al.* [60] demonstrated that etched extrusion freeformed PCL scaffolds with a roughened topography (surface roughness of up to 1.06 μm) can support hBMSC proliferation, while also inducing osteogenic differentiation, for maximal generation of bone-like tissue. The etched scaffolds induced osteogenic differentiation of hBMSCs while the un-etched scaffolds did not. Etched scaffolds also supported same levels of hBMSC proliferation as un-etched scaffolds. In addition, the hBMSCs present on un-etched scaffolds demonstrated greater migration, while hBMSCs on etched scaffolds were more rounded, indicating that surface roughness had an effect on hBMSCs morphology.

### 3.4. Mechanical Properties of the PEEK/HA Biocomposites

In both test directions the samples experienced barreling due to variation of frictional force (e.g., minimum at the center and maximum at the edges) under compressive load. It was observed that the PEEK/HA in both directions had lower yield and compressive strength and moduli than the unfilled samples. It was difficult to compare these results with previous works as other researchers have mainly studied PEEK/HA composites produced using either spherical shape HA particles or HA whisker under tensile loading [17,18,19]. Incorporation of extrusion freeformed HA continuous filaments into PEEK in this work decreased both compressive strength and moduli of the biocomposites. Similar decreasing of compressive moduli has been reported by Roeder’s group [24] for porous PEEK/HA composites. Although Roeder’s group has reported a similar effect with regard to decrease in compressive moduli, their work may not be that comparable to this study since there are some noticeable differences in the biocomposites tested; their PEEK/HA samples had 75% porosity and HA whisker with average length of 21.6 μm and an aspect ratio of 7.6 distributed randomly within the composite, while the samples tested in this study had porosity less than 5% and continuous HA filaments with 400 μm diameter and an aspect ratio of 15 with computer-controlled distribution. Furthermore, the compression molding procedures used by Roeder’s group is relatively different in terms of molding pressure and PEEK powder densification.

The samples tested in direction 2 had higher compressive modulus, yield and compressive strength than the sample tested in direction 1. It could be considered that having HA filaments oriented parallel to load axis produces remarkably higher compressive moduli and strength. This behavior has also been reported for short [62] and continuous CFR-PEEK [63]. In particular, physical inspection of the specimens at both macro and microscopic levels revealed that compression in direction 2 results in anisotropic behavior of the biocomposites. As seen in Figure 5c,d, the unfilled PEEK sample and PEEK/HA tested in direction 1 have similar strain amounts in X and Y directions, while the PEEK/HA sample tested in direction 2 have a much higher strain in direction Y than direction X (Figure 5e). Existence of the long HA fibers might be the main reason for this anisotropic behavior of the composite when tested in direction 2. PEEK needs to fracture the long HA fibers to flow in direction X, while in Y direction PEEK can flow through pores and has no need to fracture the HA structure. The side view of the sample tested in direction 2 (Figure 5f) shows the flow of PEEK between fractured HA filaments. Unfortunately, it was very difficult to use a strain gauge to measure strain in different directions due to small size of the samples. Further experiments are needed on larger specimens to enable investigation of the anisotropic behavior of these biocomposites.

As seen in Table 3, the mechanical properties of unfilled PEEK are close to that of human cortical bone which has been already reported in the literatures [11]. The PEEK/HA possesses yield and compressive strength in direction 1 within the range of cortical bone. In particular, the compressive modulus of the PEEK/HA is very close to cortical bone and this is an advantage compared to metal biomaterials. The mismatched compressive moduli of metal biomaterials with bone can cause stress shielding. For this reason metal biomaterials used must be of sufficient porosity for orthopedic application, which can add significantly to production costs and time. The use of AM systems for direct 3D printing of porous metal implant with tailored stiffness could shorten production time, yet is a relatively expensive approach. The compressive moduli of titanium alloy (Ti6Al4V) with porosity of approximately 70%, fabricated by ARCAM’s electron beam melting (EBM) AM process, was reported as 3.7–6.7 GPa that is within the range of cortical bone [64]. In contrast, the PEEK/HA composites with compressive moduli very close to cortical bone can be produced with much lower cost in a short period.

As a consequence of what was discussed, incorporation of a high HA volume into the PEEK matrix significantly affects the mechanical properties of the PEEK/HA composite. However, interconnectivity of PEEK matrix in the composites achieved through this technique is an advantage as this gives the PEEK matrix a high level of structural integrity. Therefore, a greater proportion of bioactive material can be incorporated into PEEK where required with minimum effect on mechanical properties of the composite as the PEEK matrix remains fully interconnected. Hierarchical HA scaffolds with varied pore size can be served to make functionally graded PEEK/HA composites with partially controlled mechanical and biological performances. A potential future application of these functionally graded PEEK/HA material in spinal arthrodesis is shown in Figure 6c. As demonstrated, the greatest mechanical strength is achieved in the zone where HA has larger spacing (lower HA content), and enhanced biological performance in regions in contact with bone, where HA lattice has smaller spacing (greater HA content). Computer-controlled distribution of HA in these composites ensures uniform distribution of load onto the device after implantation as well.

PEEK-based materials composed of PEEK as the base polymer with shape memory properties have been investigated [65] and used commercially (e.g., PEEK Altera^®^ by MedShape, Atlanta, GA, USA) to allow the devices to enter the target surgical site in a compact geometry and then can be triggered to deploy into the optimal geometry for fixation. The results developed in this research could contribute to the future 4D printing of devices using PEEK-based shape memory polymers with enhanced bioactivities. 4D printing has been explored in recent years for variety of applications such as printing of enhanced smart nanocomposites, shape memory polymers, actuators for soft robotics, self-evolving structures, anti-counterfeiting system, active origami, and controlled sequential folding [66,67,68]. However, 3D or 4D printing of PEEK-based materials with shape memory properties has not been reported yet.

### 3.5. Biological Performance of the PEEK/HA Biocomposites

As seen in Figure 7e, cells adhered to the HA, and a lesser extent to the rough PEEK channels (yet relatively more than the normal PEEK surface). This is an indicator that roughening of the PEEK surface can improve cell adhesion, although further investigation is required to ascertain optimum surface roughness. SEM image of samples with etched PEEK surfaces (Figure 7f) reveals a second cell localizing effect at the boundary between etched and smooth PEEK surface types. These preliminary results are consistent with various documented techniques detailing the modifying effect of PEEK surface roughness and wettability on cell adhesion and proliferation.

Sagomonyants *et al.* [69] studied machined and injection molded medical grade PEEK and CFR-PEEK together with polished, and rough medical grade titanium. According to their study, osteoblast adhesion at 4 h on injection molded variants of PEEK (Ra = 0.095 μm) and CFR-PEEK (Ra = 0.350 μm) material was comparable to polished (Ra = 0.200 μm) and rough (Ra = 0.554 μm) titanium. Osteoblast adhesion on machined samples of PEEK (Ra = 0.902 μm) and CFR-PEEK (Ra = 1.106 μm) materials were significantly less. Proliferation at 48 h determined by [^3^H]-thymidine incorporation was the greatest on the smoothest of all materials, the injection molded unfilled PEEK, which was significantly higher than the rough titanium control. The machined unfilled PEEK had the lowest DNA synthesis. They concluded that surface roughness of 0.095 μm had better adhesion and proliferation response, while for differentiation, roughness of 0.902 μm showed a better response. Recently, the effect of surface roughness of CFR-PEEK/nano-HA on *in vitro* cellular responses of osteoblast-like MG-63 cells (attachment, proliferation, apoptosis, and differentiation) and *in vivo* osseointegration was investigated [21]. Three different samples with Ra of 0.93, 1.96, and 2.95 μm were tested. The sample with moderate surface roughness (Ra = 1.96 μm) significantly increases cell attachment/proliferation and promotes the production of alkaline phosphatase (ALP) activity and calcium nodule formation compared with the other groups. More importantly, the CFR-PEEK/*n*-HA implant with appropriate surface roughness (Ra = 1.96 μm) exhibited remarkably enhanced bioactivity and osseointegration *in vivo* in the animal experiment. Surface roughness of 0.4 μm achieved in PEEK channels in this study might have different effect on cell adhesion, proliferation, and differentiation. Nevertheless, the surface morphology of the HA filament can be controlled by different sintering temperature and/or HA/TCP ration [37].

Quality control standards stipulating the testing of medical device biocompatibility require extensive investigation to confirm the true readiness for clinical application. As a pre-cursor to future development it was set out to show preliminary evidence supporting initial biological safety of the technique developed in this study. The proof of primary cell adhesion, sustained viability in contact with sample surface architecture over a seven-day period, evidence of cell bridging and matrix deposition are strongly supportive of biocompatibility. The protocol used here was only intended as an initial screening process and the authors acknowledge the depth of work yet to be completed to exhaustively document biocompatibility of novel materials. Further studies are planned to include *in vitro* cell differentiation, proliferation and molecular assays, leading to small *in vivo* models, and large animal use specific models.

## 4. Experimental Section

### 4.1. 3D Printing of Bioceramics

**Figure 8:**
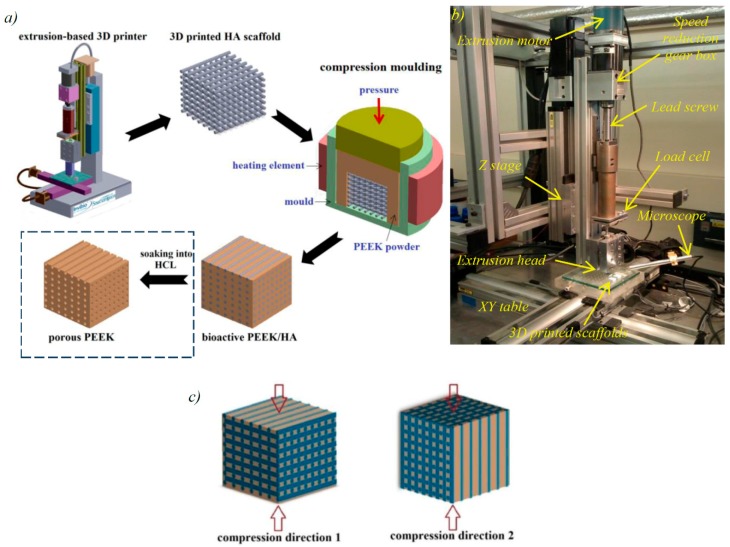
(**a**) Process steps to produce bioactive PEEK/HA composite and porous PEEK by integration of extrusion-based AM technology and compression molding process; (**b**) experimental set up for 3D printing of bioactive HA scaffolds; and (**c**) different specimen direction used for compression tests.

Figure 8a depicts workflow of the technique applied to make both bioactive PEEK/HA composite and porous PEEK structure. It comprises fabrication of porous bioactive HA scaffolds using extrusion-based AM technology, followed by PEEK melt impregnation into the HA scaffolds through the compression molding process. The PEEK/HA composite produced can be used directly with the HA bioactive phase inside. Alternatively, it can be further soaked into HCl solution to remove the HA network and produce a fully interconnected porous PEEK (shown in the dot box in Figure 8a). Clearly, when this alternative process is used to produce porous PEEK, HA can be replaced with other solvent soluble materials.

The solvent-based extrusion freeforming process, first developed by Evans and the author’s research group [37,70,71,72,73,74] was used to print highly uniform HA 3D lattice structures with controlled filament/pore size. Similar multi-nozzle extrusion method has been used for bioprinting [75]. This process involved the following steps: (i) preparation of HA paste; (ii) 3D printing; and (iii) drying, debinding and sintering of the 3D printed scaffold. The materials and procedure to make HA paste suitable for 3D printing can be found in the previous works [37,70,71,72]. An extrusion-based 3D printer was designed and built in house for 3D printing of porous scaffolds from HA paste (Figure 8b). The device comprises three-axis table (Parker Hannifin Ltd., Warwick, UK), and a stepper motor (200 steps/rev, SL0-SYN step motor, Warner Electric, New Hartford, CT, USA) driving a 2 mm-pitch ballscrew to produce a continuous force on the extrusion syringe plunger. The extrusion syringe was preloaded with the HA paste. A 100-1 reduction gearbox was used to decrease speed of the stepper motor permitting finer control of displacement. HA filaments were delivered with high precision, with diameters down to 50 μm, achieved with the use of customized nozzle and a small die land length. After 3D printing, the HA scaffolds were left at room temperature for 24 h to allow evaporation of excess solvent, and subsequently the scaffold were placed in an oven for debinding and sintering. The sintering protocol of HA was developed from the authors’ previous studies [37] in which the maximum sinter temperature was 1300 °C with a dwell time of 2 h.

Archimedes’ principle was used to measure macroporosity and microporosity of the scaffolds. The macroporosity is the porosity formed due to spacing between the filaments, which are open pores. The microporosity is the porosity inside the filament due to the not-fully-sintered HA and they are closed pores. The microporosity can be controlled by controlling different sintering temperature and/or HA/TCP ratio [37]. The details of the samples were provided in Table 1. The measuring principle, process and calculation are presented in the support materials. 

### 4.2. Production of PEEK/HA and Porous PEEK

The 3D printed HA scaffolds with different filament/pore size were over-molded with PEEK-OPTIMA^®^LT3 UF powder (Invibio Ltd., Thornton-Cleveleys, UK, used as received) through a compression molding process using static load to produce a PEEK/HA composite. A mold with an internal diameter of 25 mm was prepared from tool steel, and with a ventilation hole at size 0.5 mm on the bottom surface to avoid trapping air within the composite. The optimal molding temperature of 400 °C and pressure of approximately 0.39 MPa were determined through experimentation on over-molding HA scaffolds with external dimensions of 10 × 10 × 3 mm^3^ and different filament/pore sizes. Static loading methods were used as follows: (1) firstly the mold was heated up to 250 °C; (2) then the load was applied, and pressure was maintained until the temperature reached 400 °C; (3) the temperature and load were maintained for a further 20 min (dwell time); (4) heating was stopped, and the mold was left to cool under pressure when the PEEK matrix crystallized and solidified; and (5) composites were removed from the mold when the temperature had fallen to just below the glass transition temperature (143 °C) of PEEK, followed by cooling to room temperature, thus mitigating thermal stress and surface cracking.

Porous PEEK structures were produced by cutting off the composite from the over-molded sample to expose the HA filament, then soaking the PEEK/HA composites in HCl (37%, Fisher Scientific Ltd., Loughborough, UK) solution for 72 h. The HCL solution dissolved the HA network, leaving interconnected channels within the composite. Optical profilometry (Alicona Imaging GmbH, Raaba, Austria) was used to measure surface roughness of a typical HA filament and hollow channels following the acid treatment. To this end, two HA scaffolds with a same filament/pore size were printed, both from a same HA paste with equal solvent content, and then one of them was used to produce PEEK/HA composite, and further porous PEEK. Next, single HA filaments from the first printed HA scaffold and the PEEK channels in the porous PEEK were subjected to surface roughness measurement. Surface roughness from 5 areas was recorded and their mean was used for comparison. Profile surface measurements were performed with 100 nm vertical and 1.4 μm lateral resolutions, and were taken from a 200 μm line that was parallel to the filament and channel to minimize errors resulting from the contribution of curvatures.

PEEK/HA samples were cut at size 6 × 6 × 6 mm^3^ using diamond cutter (Mecatome T210, Presi, Brié-et-Angonne, France) and subjected to further analysis using optical microscopy (Olympus BH2-UMA, Tokyo, Japan), scanning electron microscope (SEM) (JEOL JSM-6500F, Oxford Instruments plc, Oxfordshire, UK) and computed tomography (CT) (Custom 225 kV Nikon/Metris HMX ST, Tokyo, Japan) with a resolution of 9 μm per pixel (or 9 × 9 × 9 μm^3^ voxel). CT scanning was performed to determine HA volume percentage in the composite, and to investigate fractures in the HA network and presence of air bubbles within the composites after molding.

### 4.3. Mechanical Tests

The PEEK/HA samples with average HA volume percentage of 40% (HA filament size of 400 μm and pore size 700 μm) were subjected to unconfined, uniaxial compression test using an Instron 8032 test machine at strain rate of 3 × 10^−3^ s^−1^. A 100 kN load cell was used and the test data were collected by StrainSmart software (version 6200, Vishay Precision Group, Wendell, NC, USA). Six samples were tested for reproducibility. The samples were tested in two different directions as illustrated in Figure 8c. Solid PEEK samples with 0% HA were also compression molded and tested to compare with those with 40% HA. Due to ductile nature of PEEK in compression, samples were deformed to large strains and all strains referenced in this paper are true strains (logarithmic strains). Compressive yield strength was defined as the stress after which the initial linear region deviated from linearity; yield strain was defined as the strain associated with the compressive yield strength; and modulus of elasticity was calculated from data representing the slope of the initial linear region.

Cyclic compression testing was also carried out on the specimens in direction 1 using the Instron 8032 machine operating under load-controlled mode and using sinusoidal waveform. Testing was carried out at room temperature under compression–compression mode at frequency of 5 Hz and *R*-value of 0.1 (*R* is the ratio of minimum to maximum cyclic load). The specimens were subjected to maximum stress of 20 MPa (*i.e.*, approximately 30% of compressive strength). A cooling fan was set up to avoid overheating of the samples during cyclic loading. After 1 million cycles the samples were subjected to static compression test as described earlier in order to evaluate the mechanical durability of the composite. All the PEEK/HA specimens were annealed at 200 °C to achieve a high level of crystallinity before static and cyclic compression tests using the normal protocol for injected molded PEEK parts (unfilled PEEK-OPTIMA processing guide, Invibio Ltd., Thornton-Cleveleys, UK): first, the samples were dried at 150 °C for 3 h; next the samples were heated up at 10 °C/h to reach to temperature of 200 °C and they were held for 4 h at the target temperature; then the samples were cooled down to below 140 °C with rate of 10 °C/h and the furnace was switched off allowing the samples cooled down to room temperature.

### 4.4. In Vitro Tests

After production of the PEEK/HA biocomposites it was necessary to remove any potentially cytotoxic residual manufacturing contaminates and sterilize samples prior to *in vitro* experimentation. Nine PEEK/HA scaffolds were prepared and cut using diamond cutter to approximate size, 4 × 4 × 4 mm^3^, and polished using silicon carbide papers by sequential wash, rinse and sterilisation stages. Under aseptic conditions, samples were placed in sterile 15 mL conical universal tubes (Corning Inc., New York, NY, USA), and immersed in 10 mL sterile Phosphate Buffered Saline (PBS) (Life Technologies, California, CA, USA). Samples were placed on a rotary shaker (Spiramix 10, Denley, Massachusetts, MA, USA) using 100 rotations per minute (rpm) for 10 min at room temperature, and this procedure was performed three times with fresh PBS. Samples were then washed in a 10% Triton X (Sigma Aldrich, Missouri, MO, USA) distilled water solution, at 100 rpm overnight at 4 °C. Detergent solution was rinsed 5 times using the process previously described. Following the washing process samples were placed in 10 mL sterile PBS and sterilized using exposure ultra-violet light for 6 h, with 2 h rotations. hBMSCs were isolated using standard laboratory protocols [76], and plated at density of 1 × 10^4^ onto 75 cm^2^ tissue culture flasks (Corning) in α-Minimum Enrichment Medium (α-MEM, Lonza, Basel, Switzerland), containing 10% Foetal Calf Serum (FCS, Lonza) and 1% Penicillin/Streptomycin (Life Technologies), and media changed every 3 days. Cells were passaged at 70%–80% confluence using 0.05% Trypsin (Life Technologies), re-plated onto T75 flasks, split in a 1:5 dilution and expanded in culture. Passage 3 cells were trypsinized, and suspended to achieve a 3 × 105 cells/ 100 microliters (μL) of media. Under sterile conditions, each scaffold was placed into a well of a 48-well culture plate (Corning) with the surface of interest facing upwards. Two 50-μL droplets of cell suspension were carefully applied to the surface of each scaffold with care taken to limit droplet exposure only to the region of interest. Samples were then placed incubated at 37.2 °C, 5.2% CO_2_ for 1 h to encourage cell adhesion. Following incubation 500 μL of culture medium was then added to each well, and cultured for 7 days, with media changed every 3 days. Cell viability and adherence were assessed by incubation and fluorescent imaging of Cell Tracker Green and Ethidum Homodimer (Molecular Probes, Oregon, OR, USA), respectively. After 7 days’ incubation, media was removed and samples were gently washed in PBS. Cell Tracker Green and Ethidium Homodimer solution was prepared as per manufacturer protocol, and 500 μL of it was added to each sample containing well. Samples were left to incubate for 45 min then prepared for fluorescent microscopy. Samples were washed 3 times in PBS for 5 min before fixation in 4% Paraformaldehyde (PFA) for 10 min at 4 °C. Nuclei were counterstained with a 1:1000 diamidino-phenylindole (DAPI) solution for 15 min at 4 °C, further washed in PBS and stored prior to fluorescent microscopy. Multi-layered fluorescent images were captured using an AxioCam MRm Monochrome Camera X-Cite^®^ (Zeiss, Jena, Germany) with 200 Fluorescence Microscope Light Source Leica and processed using Axiovision Software V4.0 software (Zeiss).

## 5. Conclusions

In this paper, a new technique based on extrusion freeforming and compression molding was introduced for producing bioactive PEEK/HA composite and porous PEEK. The main advantage of the technique over injection molding and compression molding is the greater control on the distribution of the bioactive phase. A static pressure of 0.39 MPa, dwell time of 20 min and temperature of 400 °C were found to be optimal for compression molding of HA scaffolds of size 10 mm × 10 mm × 3 mm with filament size of above 250 μm and pore size of above 200 μm. The technique is also an efficient method for fabricating porous PEEK with controlled porosity. In the porous PEEK, interconnected channels were formed within PEEK matrix with an average surface roughness of 0.4 μm. The PEEK/HA biocomposites produced exhibited a good biocompatibility and cell attachment, while incorporation of HA into PEEK could result in reduction of mechanical properties. The biocomposites could survive in one million compression–compression cyclic loading at 30% of their compressive strength without any degradation in compressive properties, which is quite promising for load bearing applications. 

## Figures and Tables

**Table 1:** Results of macro and micoporosity measurement for HA scaffolds with different filament and pore size.

HA Scaffold		m_s_/g	m_w_/g	V_total_/mm^3^	Macroporosity/%	Microporosity/%
Filament/μm	Pore/μm					
240	250	0.5914	0.3892	382.1	56.32	7.17
240	400	0.5701	0.3746	494.3	60.45	7.42
400	250	0.9691	0.6335	505.3	33.50	8.84
240	550	0.4668	0.3035	509.5	67.95	9.25

**Table 2:** Results of CT analysis of compression molded polyetheretherketone (PEEK)/HA composites.

HA Scaffolds		PEEK/vol. %	HA/vol. %	Air Bubble/vol. %
Filament/μm	Pore/μm			
250	200	38.6	60.3	1.1
250	200	36.5	62.4	1.1
250	250	42.4	56.1	1.5
250	250	47.6	51.6	0.8
250	400	55.9	40.9	3.2
400	250	21.3	77.7	1.0
400	400	39.4	58.3	2.4
400	550	48.7	50.1	1.2

**Table 3:** Compressive properties of the produced PEEK/HA (with 40 vol. % HA) in direction 1 and unfilled PEEK *versus* human cortical bone taken from [42].

	Human Cortical Bone		Unfilled PEEK	PEEK/HA
	Transverse	Longitudinal		
Compressive moduli/GPa	N/A	4–22	2.8	1.6–2.5
Compressive yield strength/MPa	N/A	50–200	83	54–63
Compressive strength/MPa	50–70	70–280	134	80–110

N/A: Not available.

## Supplementary Materials

Supplementary materials can be accessed at: http://www.mdpi.com/1420-3049/21/6/687/s1.
