# Supplementary material for: Characterization of New PEEK/HA Composites with 3D HA Network Fabricated by Extrusion Freeforming
Source: Molecules. 2016 May 26;21(6):687. doi: 10.3390/molecules21060687 (PMC6273399; doi:10.3390/molecules21060687)
Supplement: Supplementary file 1 [file molecules-21-00687-s001.pdf]

Supplementary Materials: Characterization of New PEEK/HA Composites with 3D HA Network Fabricated By Extrusion Freeforming

Mohammad Vaezi, Cameron Black, David MR Gibbs, Richard OC Oreffo, Mark Brady, Mohamed Moshrefi-Torbati and Shoufeng Yang

1. Calculation of Micro and Macro Porosity

The porosity measurement method was developed from [1]. To this end, the weighing pan of the balance, onto which the specimen to be measured is usually placed, has been replaced by a specially made arm from which it is possible to suspend the sample from above. The mass of the suspended HA scaffold (m_s) is measured and then the sample is placed into a small beaker of water (which does not contact the scale), which is boiled in order to evacuate air from within the internal pores of the lattice and the body of water, allowing water to fill the pores. The sample, whilst still submerged in water, is then suspended once more from the balance, and the "wet mass" of the sample (m_w) is measured (The mass that results from the sample being suspended in water). From Archimedes' principle, it is expected that this measured "wet mass" will be lower than that measured when the sample was not submerged, as the liquid will exert a force upwards against the scaffold. The magnitude of this force corresponds to the weight of the water that the scaffold displaces. Initially a cotton thread was used to suspend the sample from the scales; however, due to the absorbent nature of the thread, water saturated the thread, affecting the readings obtained from the scale. In order to prevent this, a very thin, strong and water resistant material was used. Using the data obtained from this experiment, it is possible to calculate the macroporosity and microporosity of the sample by Equation (4) and (5), respectively:

$$\mathrm{m_{dw}} = \mathrm{m_s} - \mathrm{m_w} \quad (1)$$

$$\rho_{\mathrm{water}} = 0.998\ \mathrm{g/mL\ at\ 25\ ^\circ C} \simeq 1.000\ \mathrm{g/mL} \rightarrow |\mathrm{m_{dw}}\ (\mathrm{g})| = |\mathrm{V_{dw}}\ (\mathrm{cm^3})| \quad (2)$$

$$\mathrm{V_{dw}}\ (\mathrm{cm^3}) = \mathrm{V_{af}}\ (\mathrm{cm^3}) \quad (3)$$

$$\mathrm{V_{tf}} = \frac{\mathrm{m}}{\rho_{\mathrm{HA}}} \quad (4)$$

$$\text{microporosity of filaments (\%)} = \frac{\mathrm{V_{af}} - \mathrm{V_{tf}}}{\mathrm{V_{af}}} \quad (5)$$

$$\text{macroporosity of scaffold(\%)} = \frac{\mathrm{V_l} - \mathrm{V_{af}}}{\mathrm{V_l}} \times 100 \quad (6)$$

where m_{dw}: mass of displaced water; m_s: mass of suspended scaffold; m_w: mass of wet scaffold; V_{dw}: volume of displaced water; V_{af}: apparent volume of filaments; V_{tf}: theoretical volume of filaments; V_l: total volume of scaffold (determined by measurement of external dimensions using calliper).

2. Weibull Distribution

The standard three-parameter cumulative Weibull distribution is given as [2]:

$$F(x;\ a,b,c) = 1 - e^{\left[-\left(\frac{x-a}{b}\right)^c\right]} \quad \mathrm{a,b,c} \geq 0 \quad (7)$$

where a is location parameter; b is scale parameter; and c is shape parameter. Frequently, the location parameter is not used, and the value for this parameter can be set to zero. When this is the case, $a = 0$ in Equation (7), the equation of two-parameter Weibull distribution is obtained:

$$F(x;\ b,c) = 1 - e^{\left[-\left(\frac{x}{b}\right)^c\right]} \quad \mathrm{b,c} \geq 0 \quad (8)$$

Normally, three-parameter Weibull distribution is used in conditions where the determined extreme value is not less than "a". Therefore two-parameter Weibull distribution is used to determine ultimate stress, yield stress and elastic modulus in this study. The function of *F(x;b,c)* represents the probability that the desired mechanical property (ultimate stress, yield stress or elastic modulus) is equal or less than collected data value of x. Assume a reliability function *R(x;b,c)*, and let *F(x;*b,c) + R(x;b,c*)* = 1, the reliability *R(x;b,c)* therefore represents the probability of this desired mechanical property is at least x. The equation can be written as:

$$R(x;\ b,c) = 1 - e^{\left[-\left(\frac{x}{b}\right)^{c}\right]} \quad \mathrm{b,c}\ \geq 0 \tag{9}$$

To determine parameters b and c, a linear regression method is applied. The method involves transforming Equation (8) in to the form of $e^{\left[-\left(\frac{x}{b}\right)^{c}\right]} = 1 - F(x;b,c)$. Note left hand side of the equation is the reliability function *R*(*x;b,c*). Then to take the double logarithms of both sides of the equation, the linear regression model of Y = pX + q is obtained.

$$\ln\left[\ln\left(\frac{1}{1 - F(x;b,c)}\right)\right] = c\ \ln(x) - c\ln b \tag{10}$$

The only unknown term in the equation is *F*(*x;b,c*), will be obtained by estimation from a median rank, which has the formula:

$$Median\ Rank = F(x_i;b,c) = \frac{i - 0.3}{(n + 0.4)} \tag{11}$$

This equation is also known as Benard's approximation. To calculate median rank value, the total number of data n is needed. These values are sorted from the smallest to the largest, denoting the i_{th} smallest value as x_i. For example, i = 1 for the first smallest value which corresponds to x_1, i = 2 for the second smallest value which corresponds to x_2, until the n_{th} data where $i = n$ corresponds to x_n. Then linear regression can be plotted in x-y coordinates by point (Xi,) where X = ln(x), Y = ln[ln(1/F(x; b,c))] base on Equation (10). The results are shown for plotting the regression line for ultimate stress, yield stress and elastic modulus. In order to compute *b* and *c*, a trend line is created where *c* is the slope of the trend line and *b* will be the inverse logarithm of the Y-axis intersection.

References

1. Spierings, A.B.; Schneider, M.; Eggenberger, R. Comparison of density measurement techniques for additive manufactured metallic parts. *Rapid Prototyp. J.* **2011**, *17*, 380–386.
2. Dirkolu, H.; Aktas, A.; Birgoren, B. Statistical Analysis Of Fracture Strength Of Composite Materials Using Weibull Distribution. *Turkish J. Eng. Environ. Sci.* **2002**, *26*, 45–48.
